# Barriers to cutaneous leishmaniasis care faced by indigenous communities of rural areas in Colombia: a qualitative study

**DOI:** 10.1186/s12879-022-07204-w

**Published:** 2022-03-28

**Authors:** Martha Milena Bautista-Gomez, Juliane Doerfler, Maria del Mar Castro

**Affiliations:** 1 Present Address: Centro Internacional de Entrenamiento e Investigaciones Médicas (CIDEIM), Calle 18 122-135, Campus de la Universidad Icesi (Edificio O-CIDEIM), 760031 Cali, Colombia; 2 Universidad Icesi, Calle 18 122-135, Cali, Colombia

**Keywords:** Cutaneous leishmaniasis, People-centered approach, Indigenous communities, Colombia

## Abstract

**Background:**

Neglected tropical diseases (NTDs) such as cutaneous leishmaniasis (CL) are often associated with rural territories and vulnerable communities with limited access to health care services. The objective of this study is to identify the potential determinants of CL care management in the indigenous communities in the rural area of the municipality of Pueblo Rico, through a people-centered approach.

**Methods:**

To achieve this goal, qualitative ethnographic methods were used, and a coding framework was developed using procedures in accordance with grounded theory.

**Results:**

Three dimensions that affect access to health care for CL in this population were identified: (1) contextual barriers related to geographic, economic and socio-cultural aspects; (2) health service barriers, with factors related to administration, insufficient health infrastructure and coverage, and (3) CL treatment, which covers perceptions of the treatment and issues related to the implementation of national CL treatment guidelines. This study identified barriers resulting from structural problems at the national level. Moreover, some requirements of the national guidelines for CL management in Colombia impose barriers to diagnosis and treatment. We furthermore identified cultural barriers that influence the perceptions and behavior of the community and health workers.

**Conclusions:**

While the determinants to CL management are multidimensional, the most important barrier is the inaccessibility to CL treatment to the most vulnerable populations and its inadequacy for the socio-territorial setting, as it is not designed around the people, their needs and their context.

## Background

Cutaneous leishmaniasis (CL) is the most common clinical presentation of leishmaniases, a group of parasitic diseases transmitted by sandflies, and is a Neglected Tropical Disease of the skin (skin NTD). The skin NTDs are a varied group of diseases, which are often associated with long-term disability and stigmatization and frequently affect rural and vulnerable communities with limited access to health care services. CL is reported in 92 countries, with 600,000 to one million new cases reported annually [1]. Colombia is one of the countries reporting most cases of the disease in the world [2] and has the second highest number of cases in the Americas, after Brazil [3].

Despite the high burden of CL cases, providing adequate care (diagnostics, treatment, and follow-up) remains a challenge in Colombia and the Americas. In Colombia, CL treatment is provided to patients free of charge. Most systemic antileishmanial drugs require daily administration for long periods of time and have multiple side effects [4]. However, systemic antileishmanials remain the first line of treatment, particularly pentavalent antimonials. The socio-economical setting of the population presents an additional challenge, as many indigenous communities in Colombia experience high levels of poverty, inequality, social exclusion and violence [5].

Our study was carried out in Pueblo Rico (Risaralda), a municipality with a large indigenous population located in the western Andean Region of Colombia. This area is characterized by a low population density and a high dispersion of the 83 indigenous settlements. The study area is endemic for CL and reported the fifth highest CL case rate in the country in 2019 [2]. Because of its proximity to the Pacific Choco Bio-Region, a predominantly humid and hyperhumid tropical forest prevails. In Colombia, *Phlebotomine* vectors are distributed in almost all ecological niche environments from 3500 m to sea level [4] [6]. In Pueblo Rico, *Psychodophygus panamensis* has been recently described as an important vector, whose presence overlaps with areas of higher CL case reporting [3].

The main population affected by CL are those who live in the dispersed rural area of Pueblo Rico, where 66% of people have Unsatisfied Basic Needs (UBN) [7] and limited access to health services. Many belong to the *Embera Chami* indigenous community who live in the rainforest where access to health services for CL treatment is difficult. There is a great need to explore the different barriers to access to CL diagnosis and treatment. Since data on CL in this specific population is limited, a qualitative approach can provide insight to the overall state of CL care in Pueblo Rico.

Aiming to ensure access to high-quality health care for all people, the Universal Health Coverage Forum 2017 [8] launched the people-centered health services approach. This proposes a shift in the understanding of health, with particular emphasis on the crucial role of communities in shaping health policy and health services. Instead of health systems designed around diseases and health institutions, this approach proposes health systems designed for people, organized around their comprehensive needs and including their social preferences. It is expected to contribute to achieving the sustainable development goal of Universal Health Coverage (UHC).

This research aims to identify the barriers to CL care in Pueblo Rico. Using qualitative ethnographic methods, the study applies a people-centered approach through the analysis of the following dimensions:

i. The actors: Health systems are defined by interests, values and the distribution of power among people and social groups. It is thus necessary to understand the perception of health workers and CL-affected patients [9].
ii. Health System: It is crucial to analyze a health system as complex, multifaceted and dynamic, interconnected with different sectors, actors and macro-process determinants to the access to CL treatment [10].
iii. The context: Improvements in access require an understanding of the current degree of adaptation of the CL health services delivery to their socio-political and economic context.

## Methods

### Setting

This study was carried out by investigators of the *Centro Internacional de Entrenamiento e Investigaciones Médicas* (CIDEIM) in the CL-endemic area of Pueblo Rico. Located in the Department of Risaralda (Colombia), Pueblo Rico has an area of 619.72 km^2^, most of which is a rainforest ecosystem and 99.96% which is rural. It has 13,458 inhabitants, 48.4% are *mestizos* (of mixed European, Indigenous and African ancestry), 39.5% are indigenous and 12.1% are Afro-Colombian. The indigenous population is particularly affected by high levels of poverty, social inequality, social exclusion, and violence [5].

### Study design, data collection, and participants

This qualitative study based on ethnographic methods was carried out from April to December 2019. Reporting was done according to the Consolidated Criteria for Reporting Qualitative Research (COREQ) guidelines [11]. We conducted 27 semi-structured interviews with CL patients, health workers, and community leaders in Pueblo Rico. In addition, 11 field diaries were collected through participant observation during the fieldwork.

Participants were selected through convenience sampling, using a “snowball process” according with the limitations of the area due to its remoteness and high levels of violence. The sampling ended when the information saturation point was considered to have been reached. During field visits, patients, public health workers, community leaders and health professionals who lived or worked in the rural area were directly approached. One person did not allow recording of the interview and two persons refused to participate. There was no prior relationship between the researchers and the participants. The interviews were conducted in participants’ workplaces and in public spaces. For data collection techniques, we used an interview and observation guide with the following structure: Part 1. Context, Categories: cosmovision, social fabric, territorial dynamics and social problems. Part 2. Knowledge and management of CL, Categories: previous knowledge about CL, experiences, barriers and solutions.

Interviews were conducted face-to-face by one of the authors (MMBG) and a member of the study staff (coordinator of field activities in Pueblo Rico, a registered nurse resident of the municipality with more than 10 years of experience in leishmaniasis). All interviews were conducted in Spanish, and each interview lasted between 30 and 60 min. At the beginning of the interview, the interviewer explained the general topic of the interview, shared their interest in the topic of research, and encouraged the interviewee to express ideas freely. Field notes were taken during and after the interviews. No repeat interviews were carried out. Interviews were conducted until it was determined by MMBG that the themes present in the interviews started to reoccur, which meant that data saturation was reached.

The participant observation method was developed by MMBG during the field visits to the communities, townships, informal conversations, and during meetings with social and health institutions and communities. This process was registered in field diaries and photographic records.

### Data entry and analysis

**Fig. 1:**
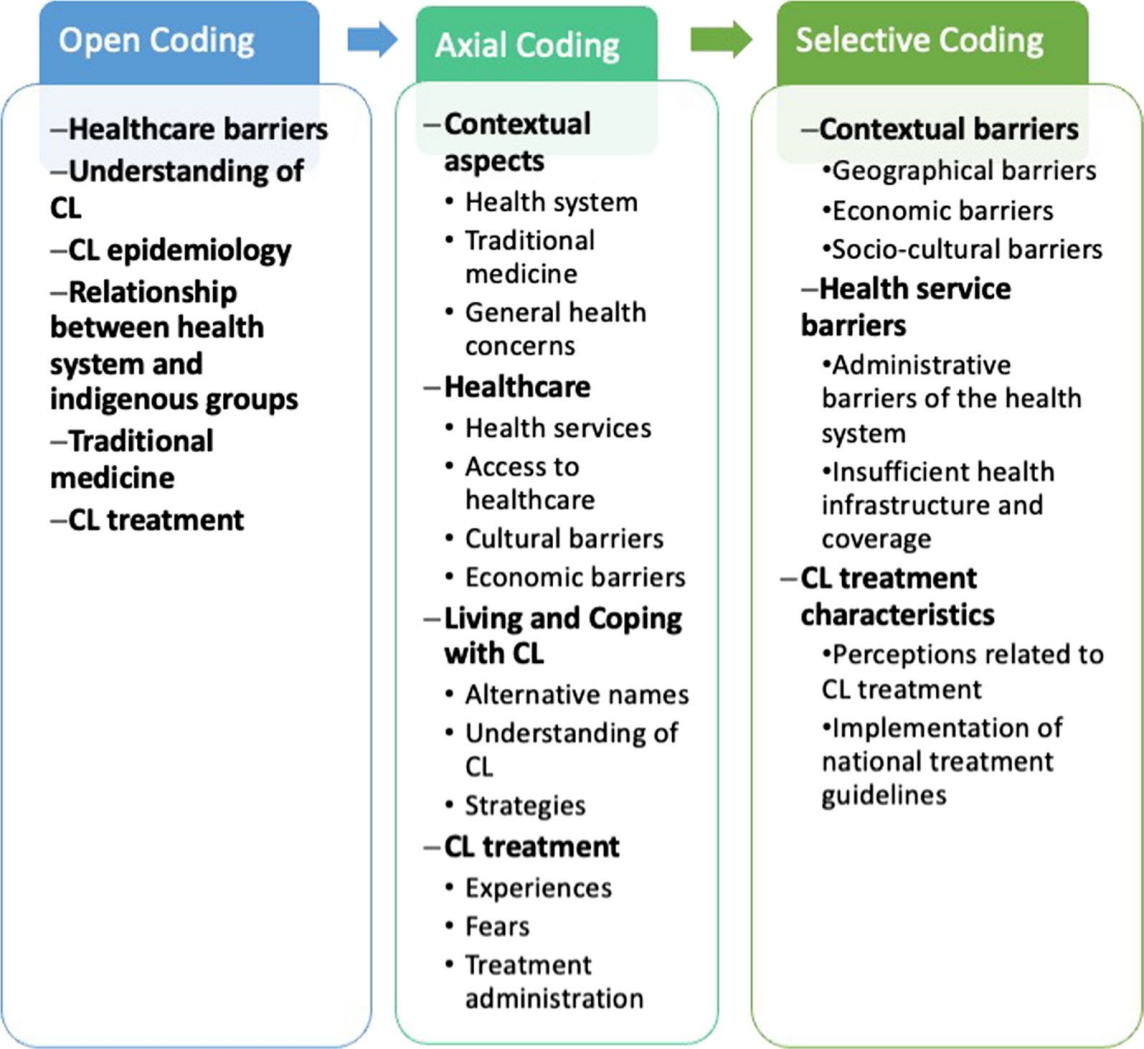
Coding process. The figure displays the coding process according to grounded theory procedures in the order of dimensions (bold font) and categories (standard font)

Audio files and field notes were transcribed and analyzed in Spanish. English translation of the relevant quotes was made at the time of writing the current manuscript. MMBG checked the transcripts and requested edits or clarifications if needed to the two research assistants that carried out the transcription. A coding framework was developed to code the material and further group it into emerging themes in an iterative manner using procedures according with grounded theory [12] following the analytical stages from open coding to axial and selective coding (see Fig. 1). N-Vivo 11 software was used for data analysis. The analysis was conducted by MMBG and MCN. The results of this study were shared with selected participants.

### Credibility, dependability, and transferability

To ensure the confirmability of the data, the coding process was carried out by two research assistants and reviewed by the researcher who led the fieldwork. To ensure the credibility of the results of the article, they were shared with a group of leaders and health workers who participated in the study. Considering the nature of qualitative research, the findings and main points of the discussion may be transferable to the analysis of health and infectious diseases in other places with comparable socio-cultural contexts facing similar challenges and issues.

## Results

We present the results of the factors that affect access to health care for CL in Pueblo Rico, Colombia. The three dimensions that define access to health in this population include (1) contextual barriers, including specific geographic, economic and socio-cultural aspects; (2) health services barriers, with factors related to administration, insufficient health infrastructure and coverage, and (3) CL treatment characteristics, which covers perceptions of the treatment and issues related to the implementation of national CL treatment guidelines (Table 1).

**Table 1:**
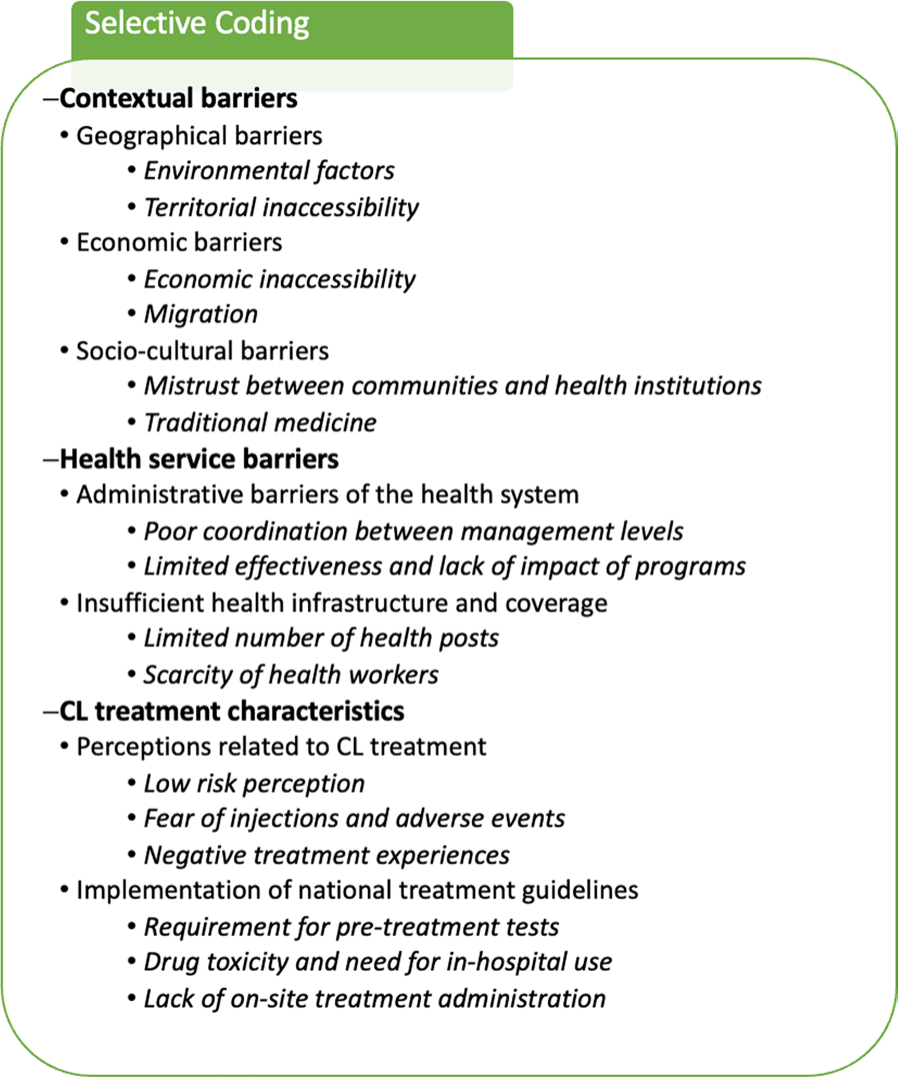
Selective coding

The table displays the results of the selective coding process in the order of dimensions (bold font), categories (standard font) and *emerging topics* (italic font)

### The environmental and geographic characteristics of Pueblo Rico favor the transmission of CL and challenge access to health services

Leishmaniasis is endemic in the research area and vector control programs recognize the presence of the vector not only in the more sylvatic areas, such as the tropical forest covering large parts of the territory, but also in the peridomicile. Traditional activities of the indigenous communities including agriculture and small-scale farming are also risk factors for *Leishmania* infection. A member of the secretary of health describes this as follows:

*“Another challenge is that the vector has adapted to the peridomicile, before it [CL] used to be more sylvatic and affected men who went inside [the forest] and now it is in the center, in some communities, as we could identify with CIDEIM in 2014 or 2015. We found many people with lesions or scars.”* (Interview 15. Member of the secretary of health. October 10, 2019).

The communities with the highest prevalence of CL live partially or totally isolated. Their villages are located in a mountainous territory with limited road communication and can only be reached by foot. Because patients must walk long distances to find informal transportation to the nearest health center, many choose not to travel. As a result, patients seek help in health centers only for very serious medical situations, as described in the following two testimonies:

*“For the case of Santa Cecilia [settlement in the Pueblo Rico municipality] it is very sui generis, because although they have a health center there, there are communities that have to walk 7 h to get to the main road and there they take transportation to be able to reach the health center, and even if they intend to take the child or not—because it can also happen—they do not have the resources to take a tuk tuk [motorcycle vehicle used for public transport] to take them there.”* (Interview 14. Health worker. October 01, 2019).

*“For example, we have a very large community, I live, for example, in Diamante, the person who is going to give me the injection lives in Minitas. I have to walk 2 h every day, one way, let’s say, 1 h going and 1 h coming, one also gets tired. I think that if there was another method, it would be easier.”* (Interview 3. Community leader. May 05, 2019).

### Indirect costs of health care: reaching the health posts and the economic loss by receiving antileishmanial treatment

Transportation and indirect costs were described as a barrier to receiving treatment and as a cause for treatment interruption. The need to travel to the health post every day for 20 consecutive days to receive antileishmanial treatment is perceived as a high burden for families with very low purchasing power. In the following quotes, a community leader and a teacher share how they experience the economic burden in conversations with affected community members:

*“I feel sorry [for the patients](…) I know a lot because I know each house, they come to me with leishmaniasis and I tell them ‘go’, [the patient says:] ‘There is no money’ (…) I give them 5000 pesos to go to Santa [Cecilia] and then they say ‘why don’t you apply it?’, [the health promoter says:] ‘No, the boss does not authorize me, I only watch and refer and bring medicine.’”* (Interview 02. Community leader. May 10, 2019).

*“(…) and the child has leishmaniasis, he has 3 strong ones, here on the hand, here in the front and on the foot (…) The father took him to the Santa Cecilia hospital, and they sent him to Pueblo Rico for an examination and the father did not take him to Pueblo Rico because of lack of resources, so at the moment he is at home. This week a lady from Santa Cecilia who works for Fundasalud [a local healthcare provider] talked to his father so that they would take him to the hospital again because he tested positive [for leishmaniasis].”* (Interview 28. College teacher. May 10, 2019).

One of the main economic activities for the indigenous population is working as day laborers, such as harvesting coffee from plantations located in neighboring towns. Working-age individuals migrate during the coffee harvest season. This often causes patients to postpone or interrupt CL treatment until the harvest is finished. (Field Diary 13. Workshop with field technicians. September 23, 2019).

### A different worldview: traditional medicine and frictions between health programs and the community

The effectiveness of health interventions is compromised by cultural frictions between health institutions and the indigenous population. Health institutions are predominantly managed by people of *mestizo* ethnicity and Afro-Colombians. Health institutions propose interventions aimed to improve health outcomes based on national guidelines that, from an indigenous perspective, often lack sense. As an example, mosquito nets provided by institutions are often used for fishing, or the use of insect repellent is promoted, but this is an expensive product that is not traditionally used by indigenous communities. Public health workers by contrast often perceive that the efforts are driven by the institutions only and lack input from the indigenous community. One community leader describes the concurrency of traditional and Western Medicine as follows:

*“(…) the council or the local governors are aware that if a disease [CL] is mild, then the traditional doctor treats it, and if it is serious it is necessary to send them to the health center and the other thing is, because we are in the community, it is distant, sometimes it takes 8 h, people do not come to the hospital to be treated for leishmaniasis or something else, there are communities that know traditional medicine, completely plant-based cures for leishmaniasis (…) those who know how to cure it, and if it is does not work, then we have to bring them to the hospital.”* (Interview 11. Community leader. September 26, 2019).

Partly due to the contextual, cultural and economic barriers mentioned above, isolated indigenous communities tend to solve health problems like CL through traditional medicine based on their ancestral knowledge. This includes ancestral doctors and healers who cure patients with medicinal plants or drinks and perform ancestral rituals. Many patients turn to traditional medicine first, and if it does not work or if complications arise, they seek help from Western Medicine. As an example, CL patients frequently attempt to heal their lesions by applying lemon, acid or hot objects. Despite the pain or potential risk of bacterial infection after chemical or physical burns of the lesions, these options are more accessible in terms of cost and time, and are more consistent with the indigenous culture. A member of the *Unidad Móvil de Salud*, a team of healthcare workers who deliver health services to distant communities, describes his perception of the cultural barriers in the following quote:

*“(…) but there is a very marked issue among the communities, the issue of traditional medicine, and it is the jaibaná [traditional healer] (…) we had a case of a girl, [the parent said:] ‘I still don’t take my girl to the doctor because I have to wait for the jaibaná at night’, I have to wait, yes, it is something they have to do, so it gets a little complicated, one would like to do things much faster, but one has to adapt with them and look for the best way, that is the work we have been doing, as I said. I know that if we go to other places, it becomes more complicated, the access, the roads, it gets a little complicated…”* (Interview 14. Health worker. October 1, 2019).

### Lack of coordination of government programs, administrative barriers of the health system and the inertia of health institutions

Administrative barriers within health institutions impede access to CL treatment. Despite numerous health and aid programs operating in the study area, we observed limited exchange of information, resources and communication among the different actors. Coordination between institutions at a national, departmental and municipal levels is often difficult.

The limited effectiveness of health programs to improve health outcomes may be attributed to guidelines which are not appropriate for the context and the territory. This is compounded by a lack of trust in the communities and a lack of commitment by some health workers to take action for change or to improve the health conditions. Health programs are often limited to compliance with national regulations and have provided the same forms of leishmaniasis control with limited effectiveness for many years. Programs do not seem to be designed with the goal of radically improving the health situation of the communities, but rather to follow administrative indicators, as expressed in the following quote:

*“(…) The chief of the reservation responds that the institutions have to know how to intervene in the communities, and if the programs do not work, it is because they do it wrong (…). Referring to the comments of the institutions on the lack of co-responsibility of the population towards the services and programs health, he says: ‘Have you come to replan the actions you do? The easiest thing is to blame others, but is that true?’ (…) He also mentions that the indigenous people have lost trust in the face of the institutionality, he says: ‘if there is trust they abide’”* (Field Diary 10. Meeting with health institutions. July 05, 2019).

Moreover, ongoing health interventions are often discontinued due to changes in political administration and thus lack long-term impact, as commented on by a community leader:

*“(…) I love [health] projects, but those projects are not for the moment, I have always been against a one-month project and that’s it, ‘we have completed the project and we are leaving’, but then there are projects that do not have an impact, they have no logic (…)”* Interview 3. Community leader. May 09, 2019).

### Insufficient health infrastructure to provide treatment to the rural population

Many of the isolated settlements are only accessible by foot, which makes it impossible for the health system to build and staff health posts. One hospital and two health posts serve an area of 19.72 km^2^ (99.96% rural and 0.04% urban [15]), while five nursing assistants are assigned to provide basic care and vaccinations for the 83 settlements [5].

Given the characteristics of anti-leishmanial treatment and the need to travel between settlements, it is not feasible for health workers to administer antileishmanial drugs to every single patient during twenty consecutive days in the vast rural area.

*“Let’s just say that there is also a lack of political will to install certain health posts that are geographically suitable for each of the communities, because only one indigenous reservation like the Resguardo Unificado [name of a reservation] has 40 settlements, so look at the magnitude of the problem, it is a matter of an administration that can generate certain resources to have much greater healthcare coverage, which is the case with the Cauca [a department in the south of Colombia with a large indigenous population] communities, which are geographically isolated, yes, but within each community there is an affordable health post people can be taken to (…)”* (Interview 14. Health worker. October 01, 2019).

Another health worker describes the difficulties arising from a scarcity of health staff to deliver antileishmanial drugs to the patients in the following quote:

*“So, I always defend my [health] assistants, for example, I say to [name of nursing auxiliary] ‘you do me a favor and apply the medicine to a man who lives in Tumacas’, but he cannot go every day from his house in Palmitas to Tumacas for 2 h to apply the medicine (…) and he has other activities. And the patient is not going to come here for 2 h if this is the closest one he has. There are nursing assistants in the community, so why don’t they apply [the antileishmanial drugs]? The auxiliary has other activities [to do] and if he also must administer the Glucantime [CL medication] to the indigenous person, it is very difficult.”* (Interview 8. Health worker. July 4, 2019).

### Negative perceptions of the indigenous population about CL treatment negatively affect treatment adherence

Health workers report that many indigenous people in Pueblo Rico are not aware of the importance of health care in general and the risk of leishmaniasis in particular. The perception of CL as a non-fatal disease leads many patients to choose to avoid or postpone medical attention. Many CL patients from the indigenous communities are also afraid of the injections, as expressed in the following two quotes by health workers:

*“The limitation with leishmaniasis is undoubtedly the treatment, with the indigenous communities we have a great barrier and that is that the indigenous people do not like injections, so they do not have continuity of treatment, they do not have any risk perception ‘if I do not have the complete treatment I will get a mucosa [mucosal leishmaniasis]’ No, for them this is not the conception, so it is very complicated.”* (Interview 15. Health worker. October 1, 2019).

*“(…) people are very afraid of that shot, again I insist and there are so many (…) people do not finish the treatment, because there are so many shots, people start very conscientiously until halfway through, from there they start to decrease, they leave and they leave (…) people are very afraid (…)”* (Interview 08. Health worker. July 04, 2019).

Some indigenous patients are skeptical about the effectiveness of the CL treatment due to their experiences with treatment failure. In addition, many are afraid of adverse effects of the CL treatment, such as vomiting, diarrhea, muscle cramps or headaches. In an informal conversation, a patient mentioned that he could live normally with the CL lesion, but he could not work because of the side effects of the CL treatment. The treatment has to be administered by trained personnel to avoid further side effects, as stated by this health worker:

*“(…) the volume [of Glucantime] is very large, it is necessary to administer 15 ml daily in the gluteus of the patient. Then on the third day (…) the gluteus is already edematous, and if the person has a bad technique in the application, it can cause complications (…)”* (Interview 8. Health worker. July 4, 2019).

### Treatment characteristics, requirements and unavailability pose additional challenges to patients and healthcare providers

Another barrier to leishmaniasis care is the requirement for diagnostic and pre-treatment laboratory tests. According with the national guidelines for leishmaniasis management in Colombia [16], these tests are mandatory to assess eligibility for systemic treatment. This is due to the potential toxicity of antileishmanials such as meglumine antimoniate or pentamidine. Likewise, the diagnostic process is also a barrier. In the case of Pueblo Rico, blood samples are taken twice a week at the health posts. They are then processed at a secondary health care level in neighboring town. The turnover time of the results is approximately 15 days.

In the department of Risaralda, CL treatment was previously administered in the indigenous communities through health promoters. Today however, this is not possible due to the lack of availability of rural staff. Moreover, considering the risks of adverse events, the National guidelines limit CL treatment to In-Hospital Use only. In Pueblo Rico, CL treatment is currently provided at the hospital and the two health posts of the municipality. During a workshop with health workers, they raised the following possible solutions to the problem of treatment distribution:

- *Establishment of health posts closer to the communities to mitigate territorial access barriers*
- *Training of community members to administer the drug in communities*
- *Ensuring sufficient health staff to administer the treatment in the communities*
- *Increase the use of pentamidine or miltefosine, which unlike Glucantime are administered for only 5 days, providing accessibility to the community*

(Field Diary 13. Workshop with field technicians. September 24, 2019).

### Feedback to the results from members of the community

Findings were presented and discussed with members of the community and leaders in a meeting conducted by MMBG. Overall, participants agreed with the findings (in a Spanish version) as presented in the current study, a summary of their feedback and some of their comments to the results are presented in the Table 2.

**Table 2:** Summary of the feedback from the community and study participants

Findings	Feedback (notes from the discussion and quotes)
The environmental and geographic characteristics of Pueblo Rico favor the transmission of CL and challenge access to health services	Leishmaniasis is always present in the municipality, so it is important to control it.
Indirect costs of health care: reaching the health posts and the economic loss resulting from antileishmanial treatment	During the time of treatment, there are considerable costs to the families. For example, when a child receives treatment, the mother must travel with the child during the 20 days it lasts, plus the time it takes for the diagnosis. This means that to bring one kid, mothers must leave their other children alone at home, and one has to bear in mind that they [the indigenous] are usually very large families.
A different worldview: traditional medicine and frictions between health programs and the community	For the indigenous population, the bites of the sandflies are normal and that is why measures that are within their reach must be implemented. *For example, previously the indigenous people chewed tobacco, and this was smeared on their skin as a barrier or protection measure against bites* (Community leader, Santa Cecilia).
Lack of coordination of government programs, administrative barriers of the health system and the inertia of health institutions	The bureaucratic problems mean that the health worker is hired very late and has very little time to develop their activities, therefore, they end up executing tasks just to fulfill their work, but without the sense of belonging that it takes.
Insufficient health infrastructure to provide treatment to the rural population	The deficiency is mainly in the rural area. "*There is a medical service, but you have to see how many turns [‘vueltas’ a colloquial term to indicate visits or administrative procedures] a person has to make to be treated*" (Member of the community, living in Santa Cecilia)
Negative perceptions of the indigenous population about CL treatment negatively affect treatment adherence	The first option, from the cultural perspective, are the Jaibanás [traditional healer]. People do not like injections and are afraid of the adverse reactions.
Treatment characteristics, requirements and unavailability pose additional challenges to patients and healthcare provide	There are few alternatives to treatment. Participants point out the importance of differential health interventions, and although they recognize that there is a policy for indigenous health, they affirm that it does not work in practice. They propose to adopt differential control measures, to guarantee the attention of infectious diseases in the indigenous population.

## Discussion

### Structural barriers: the problems of rural territories and public policies significantly affect the operation and access to LC treatment

According to the framework of social determinants of health**,** structural factors have a broad impact on access to health [17]. Most of the barriers to accessing CL treatment at a local level result from structural problems. These include the long distances the population travels to health centers in urban areas, the limited availability of staff and facilities as well as the poor infrastructure of health services. These structural problems negatively affect access to health, particularly for populations living in rural areas, which are largely affected by CL. In Colombia, 80.8% of the 5025 reported cases in 2020 were from dispersed rural areas. Poverty, social inequalities, armed conflict, violence, political centralism and poor infrastructure [18] pose additional barriers to accessing health services.

In rural areas in Colombia, 45.7% of the population live in poverty [19]. Although treatment costs for CL are covered by the Colombian health plan, rural populations still face challenges in accessing treatment. Their low purchasing power often leads to an inability to afford out-of-pocket expenses, such as transportation and per diems. Additionally, there are opportunity costs like the loss of income due to absence from work. This situation is not limited to Colombia [20, 21]. In a recent study on CL among indigenous groups in Bolivia, participants described expenditures that corresponded to 600 USD, which is more than twice the monthly minimum wage in the country [22].

Limited availability of human resources to serve these communities was evidenced by this study. This phenomenon is not limited to our research area. Despite the high burden of infectious diseases, there is a deficit of human resources to provide care in rural areas in Colombia. There are only 28.8 health professionals/10.000 inhabitants of dispersed rural areas, compared to 102 professionals/10.000 inhabitants in urban centers [23].

With respect to public policies, Colombia faces structural problems related to political clientelism, corruption and an inefficient bureaucratic system especially in the most marginalized areas of the country [24]. Most of these factors explain the administrative health services barriers identified at a local level such as: the disarticulation between health institutions, the discontinuity of health programs and projects, the challenges related to available budget, and the delays in hiring staff (approximately the first 3 months of the year there is no permanent health staff), among others.

### Colombian health system barriers: the regulation and availability of CL treatment makes it inaccessible to the most vulnerable population and inadequate for the socio-territorial context

Although the requirements of national guidelines for leishmaniasis management in Colombia are based on the potential drug toxicity, most of their measures are not feasible in dispersed rural areas such as Pueblo Rico. This includes availability of diagnostic tests and laboratory testing (e.g., creatinine, amylase, etc.) pre- and during treatment which are recommended to monitor safety, as well as the administration of the antileishmanial drugs in a health facility. Although their primary aim is to improve the safety of antileishmanial treatment, these regulations often impose barriers to treatment of CL due to insufficient health centers and lack of staff and laboratory capacities for test analysis.

The lack of adequate health infrastructure in Pueblo Rico highlights the need to explore new strategies to administer CL drugs inside the communities. It is necessary to explore new mechanisms that promote equitable access and inclusiveness of health, developed in similar contexts for other diseases that could be replicated to improve access to treatment. These new approaches include home visits by the health team to remote rural communities [25, 26] or capacity building programs, like the training of community representatives in indigenous communities in the Peruvian amazon [27]. It is also important to observe the different forms of implementation of treatment guidelines. In our prior unpublished work, we observed that in another Colombian municipality, health institutions have tried to adapt the national regulations to their context, with pre-treatment exams only being applied to those over 40 years of age (a practice likely based on a previous version of the Colombian treatment guidelines, which indicated pre-treatment laboratory testing in patients ≥ 45 years of age). They furthermore had nursing auxiliaries from the hospital administer the treatment in the rural villages during weekdays. Additionally, it is important to consider the social and institutional capital of the area. There are a total of 27 health assistants for the rural areas, including vaccinators, health promoters and nursing assistants from the four health institutions. In addition, most rural communities, even the most remote, have members with training in health (nursing auxiliaries).

Moreover, new alternatives for diagnosis and treatment are urgently needed for CL. Current treatments are costly, long, and often involve drugs with a high level of toxicity that can potentially have severe side effects. There is a need for rapid diagnostic tests for an early detection of CL cases as well as an easy-to-administer oral or topical treatment, as mentioned in the new NTD roadmap elaborated by WHO One of the critical actions of the 2021–2030 roadmap is to “Develop and scale up easy-to-administer oral or topical treatments that could be used in health centers”. This aspect is relevant for the development of Target Product Profiles (TPP) that guide research and development of new drugs. For instance, the Drugs for Neglected Diseases Initiative (DNDi)’s TPP for CL treatments include local therapies, with no contraindications or that can be assessed at a primary health care level, among other desired characteristics [28]. Due to their good risk–benefit ratio, local therapies have been recommended by WHO since 2010 for patients who meet specific clinical criteria. In the Americas, local therapies were also included in PAHO 2013’s treatment recommendations, but their adoption is limited. These alternatives need to be more widely implemented, made affordable and available to CL patients.

### Cultural barriers: cross-cultural conflicts lead to negative perceptions and behaviors that are critical to the decision to access health services for CL treatment

Cross-cultural conflicts often arise from the lack of recognition of different forms of health knowledge and practices and the ways in which these, including scientific medical knowledge, are influenced by different cultural backgrounds. When this is the case, bottlenecks often occur that do not allow for solutions to the central problems [29]. The cultural misunderstandings in Pueblo Rico are evident in the frictions between Western and traditional medicine and the low level of agreement between the community and the health actions of the institutions.

As has been documented in other studies, cultural factors frequently condition the system of perceptions and behaviors of each cultural group in relation to the use of health services. A Colombian study for example reported a scarce use of medical services due to low trust in the quality of the primary care services [30]. In Pueblo Rico, the community was perceived to have little concern for improving their own health. This behavior can be related to the low-risk perception of leishmaniasis and the distrust in institutions. The community however see the institutional behavior as limited to compliance with health regulations, which in turn may be related to their perception that their work is constrained by the community and the political structures.

Cultural discrepancies constitute an important barrier to access health care. Given that health programs are designed, implemented and incorporated by people (health workers and patients), the interest and willingness of all actors are fundamental to imply change to improve the current CL care management. There is an urgent need for institutions to promote creative ways to guarantee access to leishmaniasis treatment appropriate to the context. Likewise, it is important to stress that communities are co-responsible for their health and are willing to learn and lead change processes. In the case of Pueblo Rico, both set of actors bear responsibility.

### Considerations and limitations

Data collection for the current study was limited to urban areas and nearby villages located approximately 2 h from nearest urban area. This was due to the difficulties to reach more remote villages, which are up to 8 h away from the nearest urban area and are affected by the presence of non-state armed actors. Sample size was limited by the distrust and unwillingness of indigenous people to participate in the interviews due to the violence in the area and closed traditional communities. Furthermore, communication barriers limited the depth of the interviews. Even though most of the population’s members speak Spanish, some showed poor comprehension of the questions and little concordance and fluency in their answers.

In contrast, participant observation constituted a very important source of data. The regular frequency of visits to some communities, the contact with people in their natural living spaces and the informal conversations and participation in community activities provided an understanding of the context that was crucial for the data analysis.

## Conclusion

The determinants to CL management are multidimensional, involving structural, cultural and health system barriers. It is thus necessary to pursue a comprehensive approach to improve the accessibility of CL care. Our results suggest that the most relevant barrier for CL care is related to the administration of the treatment and type of antileishmanial drug. Since the treatment is designed around the disease and health institutions rather than the affected people, it is inaccessible to the most vulnerable population and inadequate for the socio-territorial context.

Similar to the findings of other studies on CL and indigenous populations [22, 31], we found that the structural inaccessibility to CL treatment in Pueblo Rico due to cultural, geographic, economic and health system barriers causes patients to turn to traditional medicine before seeking help in Western medicine. However, this can also lead patients to wait until the disease reaches an advanced stage before seeking treatment, which in turn can affect the effectiveness of the treatment.

## Data Availability

The data presented in this study are available on request, subject to authorization by the CIDEIM ethics committee. Requests can be sent to the corresponding author and to the institutional Ethics Committee of CIDEIM at: cideim@cideim.org.co.

## Abbreviations

NTD: Neglected tropical diseases
CL: Cutaneous leishmaniasis
COREQ: Consolidated criteria for Reporting Qualitative research
UHC: Universal Health Coverage
IRB: Institutional Ethical Review Board
CIDEIM: Centro Internacional de Entrenamiento e Investigaciones Médicas
DNDI: Drugs for neglected diseases initiative
WHO: World Health Organization
SIHI: Social innovation in health initiative
TDR: Special programme for research and training in tropical diseases
UNICEF: United Nations International Children’s Emergency Fund
UNDP: United Nations Development Program
UBN: Unsatisfied basic needs
